# Ecology, Biology, Environmental Impacts, and Management of an Agro-Environmental Weed *Ageratum conyzoides*

**DOI:** 10.3390/plants12122329

**Published:** 2023-06-15

**Authors:** Amarpreet Kaur, Shalinder Kaur, Harminder Pal Singh, Avishek Datta, Bhagirath Singh Chauhan, Hayat Ullah, Ravinder Kumar Kohli, Daizy Rani Batish

**Affiliations:** 1 Department of Botany, Panjab University, Chandigarh 160014, India; amanhayer411@gmail.com (A.K.); daizybatish@yahoo.com (D.R.B.); 2 Department of Environment Studies, Panjab University, Chandigarh 160014, India; hpsingh_01@yahoo.com; 3 Department of Food, Agriculture and Bioresources, School of Environment and Resource Development, Asian Institute of Technology, Klong Luang, Pathumthani 12120, Thailandhayatbotanist204@gmail.com (H.U.); 4 The Centre for Crop Science, The University of Queensland, Gatton, QLD 4343, Australia; b.chauhan@uq.edu.au; 5 Amity University, Mohali 140306, Punjab, India; rkkohli45@yahoo.com

**Keywords:** agricultural weed, Asteraceae, billy goat weed, ecological impacts, environmental weed, invasive weed, weed management

## Abstract

*Ageratum conyzoides* L. (Billy goat weed; Asteraceae) is an annual herbaceous plant of American origin with a pantropical distribution. The plant has unique biological attributes and a raft of miscellaneous chemical compounds that render it a pharmacologically important herb. Despite its high medicinal value, the constant spread of the weed is noticeable and alarming. In many countries, the weed has severely invaded the natural, urban, and agroecosystems, thus presenting management challenges to natural resource professionals and farmers. Its interference with agricultural crops, grassland forbs, forest ground flora, and its ability to replace native plant species are of serious concern. Therefore, it is pertinent to monitor its continuous spread, its entry into new geographic regions, the extent of its impact, and the associated evolutionary changes. While management strategies should be improvised to control its spread and reduce its adverse impacts, the possible utilization of this noxious weed for pharmacological and agronomic purposes should also be explored. The objective of this review is to provide a detailed account of the global distribution, biological activities, ecological and environmental impacts, and strategies for the management of the agro-environmental weed *A. conyzoides*.

## 1. Introduction

*Ageratum conyzoides* L. (Family Asteraceae) is an aromatic annual herb native to Central and South America [1]. The genus “*Ageratum*” refers to the Greek term “*ageras*”, signifying the seemingly non-ageing quality of this species (referring to its long lifespan), and the species epithet “*konyz*” refers to the Greek name of the plant species, *Inula helenium*, which the weed resembles [2]. The common name, “goat weed” or “Billy goat weed”, is derived from an Australian male goat due to a close resemblance in odor [3]. It has two subspecies: “*latifolium*”, found within the American continent, and “*conyzoides*”, with a distribution throughout the tropical and subtropical regions of the world [2].

The plant was initially distributed across different continents owing to its ornamental value, but it has now naturalized and spread in nearly all types of ecosystems, colonizing aggressively, and presenting management issues to environmentalists, ecologists, conservation managers, and agronomists [4]. Apart from its invasive abilities, the plant is well known for its strong phytochemical composition, unique biological attributes, and versatile applications in agriculture and industry. While previous reviews regarding *A. conyzoides* primarily emphasized its potential as a medicinal [5] or industrial [6] crop, this holistic review aims to provide a succinct overview of its global distribution, biological activities, ecological and environmental impacts, and management strategies; thereby encompassing all facets of the plant’s behavior. The review serves as an updated introduction to *A. conyzoides*, while highlighting the fields where most of the current studies are focused and identifying the research areas requiring further attention.

## 2. Global Distribution

**Figure 1:**
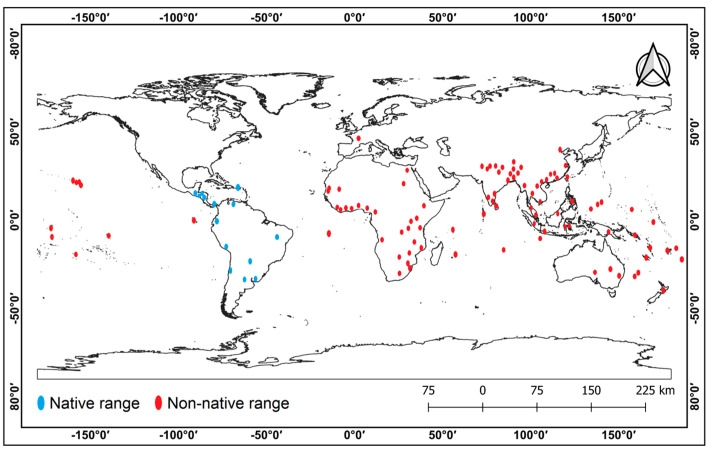
Distribution of *Ageratum conyzoides* in native and non-native regions across the globe (Source: Vélez-Gavilán [7]).

The occurrence of *A. conyzoides* as a troublesome weed came to attention between 1960 and 1980 [7]. Many reports suggest its widespread distribution in various countries in Asia, Africa, Australia, and America (Figure 1). In Asia, its invasion has been reported in India, Malaysia, the Philippines, Malawi, Cambodia, Thailand, Vietnam, Bangladesh, Pakistan, Sri Lanka, Indonesia, China, and Japan [4,8,9,10,11,12,13,14]. In Africa, it is widely distributed in South Africa, East Africa, Zimbabwe, Mauritius, Angola, Ethiopia, Kenya, Liberia, Tanzania, Uganda, the Democratic Republic of the Congo, Egypt, Ghana, and Nigeria [9,11,15,16,17,18,19,20]. The herb is also found in Australia [21], Fiji, New Caledonia, the Cook Islands, the Solomon Islands, Vanuatu [8], the Mariana Islands, the Hawaiian Islands, the Virgin Islands, the Galapagos Islands [14], the Federated States of Micronesia [22], and Palau [23]. *A. conyzoides* has considerably enhanced its distribution range in the last few decades, and research utilizing ecological niche models suggests that the weed has the potential to perform better under climate change scenarios and to expand into uninvaded regions in the future [1,24,25]. A detailed account of its distribution on different continents and island ecosystems around the world is provided in Figure 1.

## 3. Ecology

**Figure 2:**
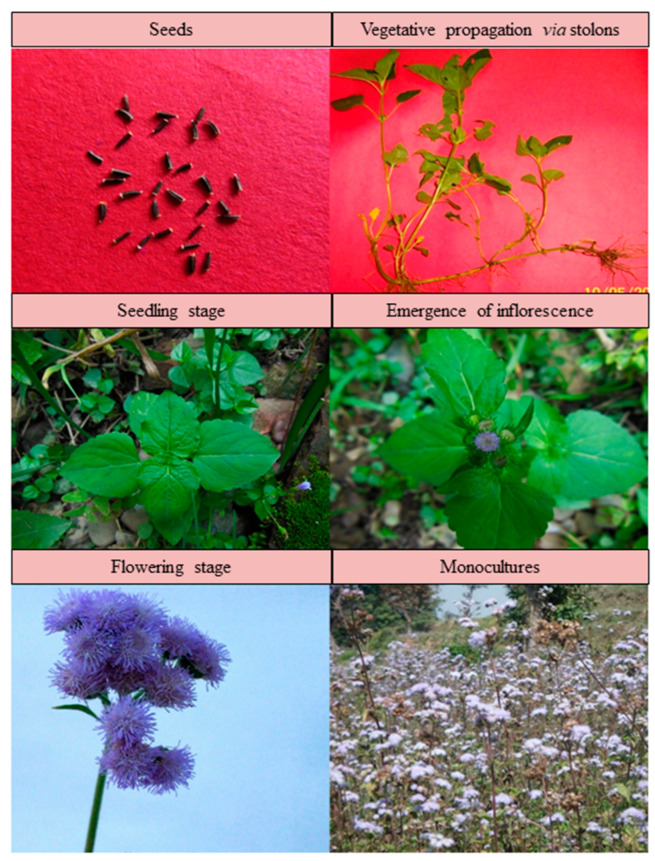
Different growth stages of *Ageratum conyzoides*.

**Figure 3:**
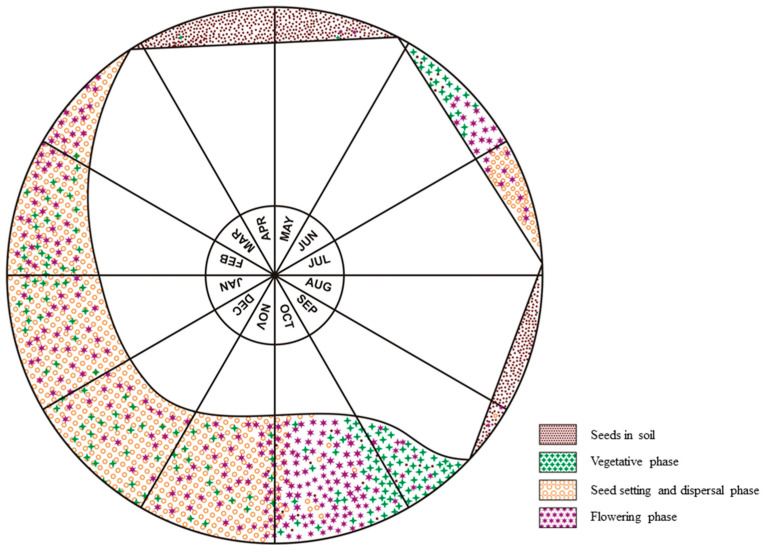
Life cycle pattern of *Ageratum conyzoides* in Chandigarh, India (Source: Arora [27]).

*Ageratum conyzoides* grows erect up to 1 m with profuse branching, and is characterized by oval, pubescent leaves with toothed margins and a reddish stem [1] (Figure 2). White to mauve-colored hermaphroditic disc florets are arranged closely in the inflorescence, i.e., the capitulum [1]. The variations in flower color are associated with different chemotypes of the plant [26]. The seeds bear an aristate pappus that facilitates anemochory in the plant [13]. Vegetative reproduction occurs through stolons [4] (Figure 2). In the northern plains of India, *A*. *conyzoides* appears twice annually, with a short (June–July) and a prolonged (October–March) life cycle [27] (Figure 3). *A. conyzoides* has attained the status of a noxious biological pollutant, especially in croplands, by virtue of many ecological and biological traits that help in its successful invasion in alien environments.

### 3.1. Environmental Suitability and Adaptability

*Ageratum conyzoides* is highly adaptable to different temperatures, moisture conditions, soil textures, and altitudinal ranges. Its growth is best suited to temperatures ranging from 20–25 °C, but it also survives well at 15–30 °C [27]. This explains its prevalence at higher altitudes (i.e., temperate climates) as well as on the plains (i.e., tropical climates) [28]. Its growth is not affected by soil fertility status, and the weed acclimatizes well to high light intensity and severe salinity stresses [29]. High phenotypic plasticity allows the plant to settle in novel surroundings via suitable biomass allocation [30].

### 3.2. Ecological Range

**Figure 4:**
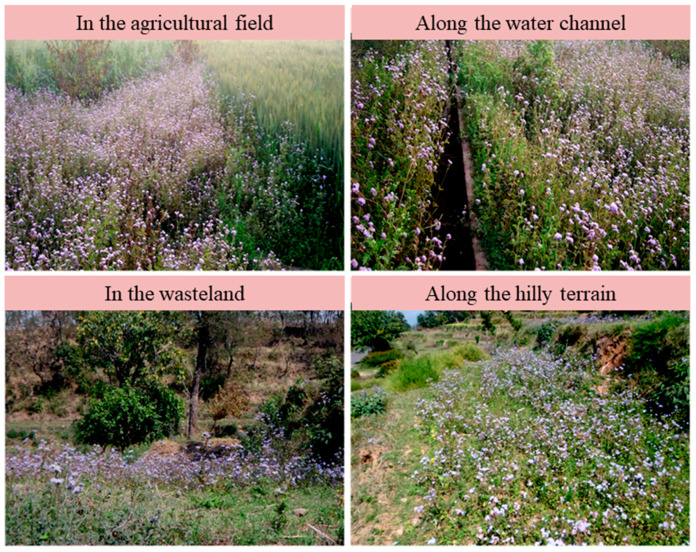
The spread of *Ageratum conyzoides* in various habitats.

Within its natural geographic range, *A*. *conyzoides* is only considered to be an agricultural weed, but in invaded areas, its thickets can be spotted in agricultural lands, grasslands, wastelands, natural forests, wetlands, plantations, vegetable gardens, pastures, orchards, tea plantations, alongside water channels, disturbed sites, sites of fresh landslides, and roadsides [4,31] (Figure 4). The plant is an early colonizer of abandoned fields or shifting cultivation sites, and sometimes it dominates as a pioneer community [32]. It can easily colonize available gaps in widely spaced annual crops or plantations [2,33].

### 3.3. Reproductive and Regenerative Potential

Fast growth, a short life cycle, early reproductive maturity, prolific seed production, and vegetative reproduction enable *A*. *conyzoides* to establish itself in an alien environment [2]. The large production of small, lightweight seeds with a wide range of dispersal enables its fast spread and colonization [13]. A study conducted in China revealed that *A*. *conyzoides* dispersed at a minimum speed of 2.4 km year^−1^, mainly through human or wind-mediated dispersal [13]. A single plant is reported to produce 40,000–95,000 seeds with a germination rate of 50% [34]. Additionally, the weed may also proliferate quickly through vegetative reproduction by stolon production [4].

### 3.4. Allelopathy

Aqueous extracts, volatile oils, and the rhizospheric soil of *A*. *conyzoides* are known to possess allelochemicals that interfere with the growth and development of associated plants [35,36]. The germination, plumule, and radicle length of several crop species were reported to be affected by *A*. *conyzoides* [36,37,38,39]. In addition, the large amounts of weed residue left at the infestation sites interfered with the growth of the succeeding crops [40]. Allelochemicals are released from the plant by leachates from foliage and plant litter by rain, volatilization through aerial parts as root exudates, or the decomposition of organic matter. The mechanism behind the phytotoxic effect of these compounds has also been examined. It was found to alter the actions of the respiratory enzymes [41] and the growth hormone gibberellic acid, which is involved in seed germination [42]. However, further investigations are required to accurately identify its mode of action.

In addition to the above-mentioned characteristics, several other factors such as morphological and phenological adaptations, a short juvenile phase, long flowering and fruiting periods, the absence of natural enemies (pests, pathogens, and herbivores), the resistance to native predators due to the release of a wide array of secondary metabolites, and unpalatability due to the highly phytotoxic nature of the plant contribute to its unchecked prevalence in the invaded ranges [2,4]. In general, wide adaptability in invasive species has been reported to give rise to intraspecific variations that maximize their fitness under heterogeneous environmental conditions [43,44]. The presence of different chemotypes, ecotypes, and biotypes in *A. conyzoides* [5,26] indicates its ability to expand extensively across diverse geographical regimes by overcoming its physiological limitations and environmental barriers.

## 4. Biological Activity

The weed has been used for a wide range of biological activities since antiquity, as it possesses a raft of miscellaneous chemical compounds. The natural product chemistry and biological properties of *A. conyzoides* have been extensively investigated. Some of its prominent biological activities and potential applications are discussed further.

### 4.1. Natural Product Chemistry

A wide variety of secondary metabolites have been identified in the aqueous extracts and volatile oils obtained from various parts of *A*. *conyzoides* [3]. The essential oil contains phytochemicals such as phenols, phenolic esters, and coumarins, whereas the other parts of the plant contain terpenoids, steroids, chromenes, pyrrolizidine alkaloids, and flavonoids [5,45]. As per Dores et al. [46], the weed contains the maximum phenolic compounds in its roots (23 mg mL^−1^), followed by flowers (19 mg mL^−1^), and leaves (15 mg mL^−1^), while the maximum content of flavonoids is observed in leaves (5.7 µg mL^−1^), followed by flowers (5.4 µg mL^−1^) and roots (4.8 µg mL^−1^). The essential oil of *A*. *conyzoides* constitutes nearly 200 chemical compounds, of which precocene I and II, their derivatives, monoterpenes, and sesquiterpenes are the major constituents, accounting for 77% of the oil [35,47].

### 4.2. Pharmacological Properties

*Ageratum conyzoides* has ethnobotanical importance due to its use in traditional medicinal practices [5]. Leaves of the plant are commonly applied to heal burns and wounds across the world and are used in ayurvedic medicines prepared for fever, earache, cold, headache, rheumatism, diabetes, infertility, blood clotting, diarrhea, ear infections, etc. [3,48]. According to the Bodo community of Assam, the root extracts of *A*. *conyzoides* help fight malaria [49]. A recent study has shown that the leaves of *A*. *conyzoides* possess alpha-amylase inhibitory potential, which is beneficial in treating type II diabetes as well as its secondary complications, by lowering postprandial hyperglycemia [50,51]. Antitumor and co-chemotherapeutic effects of *A*. *conyzoides* have also been reported [52,53]. The correlation between in silico and classical pharmaceutical investigations implies that the antidiabetic and anticancerous activities of the weed stem mainly from chromenes (precocene I, precocene II, and VMDC) and sterols (betasterol and stigmasterol), respectively [45]. *A. conyzoides* has also proven to be a potential adjuvant agent in the treatment of polycystic ovary syndrome [54]. Extracts of the weed also exhibited antibacterial activity by inhibiting 40.4% of the 464 strains of drug-resistant gram-positive and gram-negative bacteria [55]. Crude extracts of the plant severely affected the flagella and ventral discs of *Giardia duodenalis* (the causal organism of giardiasis in humans), thus impeding their ability to attach to the surface of mucosal cells [56]. Phyto-formulations based on *A*. *conyzoides* possess acaricidal potential against acaricide-resistant ticks, infesting cattle, and buffalo [57]. It is suggested that the compounds isolated from *A*. *conyzoides* can combat numerous pathogenic strains that cause infections in humans and livestock [55].

### 4.3. Insecticidal Properties

The essential oil obtained from *A*. *conyzoides* can kill insects by modifying their digestive systems. Precocenes present in the essential oil possess antijuvenile hormonal activity, causing precocious metamorphosis in insects [3]. The oil may also induce abnormalities at the phenotypic or genotypic level in the larvae of *Aedes*, *Anopheles*, and *Culex* spp. [58]. The weed has also shown promising results against plant and animal pests such as *Rhipicephalus microplus*, *Phytophthora megakarya*, *Diaphania hyalinata*, *Tribolium castaneum*, *Helicoverpa armigera*, *Plutella xylostella,* etc. [11,59,60,61,62,63] indicating the plant’s potential to be utilized for controlling insect pests.

### 4.4. Fungicidal Properties

The essential oil of *A*. *conyzoides* can serve as a substitute for synthetic fungicides due to its strong fungicidal and aflatoxin-inhibitory potential [64]. Flavones released by the weed showed results equivalent to those of the commercial fungicide carbengin by reducing fungal infections in citrus plantations [65]. Essential oils and extracts of the weed showed very strong antifungal activity against *Drechslera* sp., *Puccinia arachidis*, *Botryodiplodia theobromae*, *Fusarium verticillioides*, *Alternaria cucumeria*, *Curvularia lunata*, *Pyricularia oryzae, Rhizoctonia solani*, *Aspergillus flavus*, etc. [66,67,68,69,70], thereby providing an effective and eco-friendly solution for the management of fungal pathogens.

### 4.5. Herbicidal Properties

*Ageratum conyzoides* has recently been recognized as a novel agrochemical tool for weed control. Plant extracts hindered the growth of *Digitaria sanguinalis*, *Lactuca sativa*, *Amaranthus caudatus*, *Amaranthus spinosus*, *Echinochloa crus-galli*, *Monochoria vaginalis*, and *Aeschynomene indica* [71,72,73]. When intercropped in citrus orchards, it significantly inhibited weeds such as *Cyperus difformis*, *Bidens pilosa*, and *Digitaria sanguinalis* [74]. Application of the dried leaves of *A. conyzoides* killed 75% of the weeds present in the rice field and increased the grain yield by 14% compared to a commercial herbicide [72]. In another study, the extracts of *A. conyzoides* showed promising results in controlling weeds while at the same time conserving and increasing the soil microflora [75], thereby indicating its beneficial use as a bioherbicide.

*A. conyzoides* show promise as a valuable biosource for developing effective formulations for clinical, industrial, and agricultural uses. However, further scientific research is necessary to assess the chronic toxicological reactions, potential side effects, safe dosage levels, and long-term interactions and feedback [69]. A recent study indicated that the removal of pyrrolizidine alkaloids plays a significant role in determining the toxicity of *A. conyzoides* extracts [76]. This finding underscores the importance of considering and managing the presence of these compounds in formulations and products derived from the plant. Furthermore, there is a need for advanced molecular technologies, including RNAi, CRISPR/Cas9, multi-omics approaches, etc., which may aid in deciphering the action mechanisms and enhance these formulations [77]. It is not only vital for enhancing the economic value of *A. conyzoides* but also for developing sustainable and safe strategies for biomedical, environmental, and agricultural applications.

## 5. Ecological Impacts

*Ageratum conyzoides* damages ecosystems both economically and ecologically, either directly competing with the native plants for resources, and/or indirectly by altering ecosystem processes and ecological functioning such as soil nutrient cycling, pollination, etc. Nevertheless, there is a scarcity of research in this domain, specifically in terms of studies that offer empirical data to accurately assess the magnitude of the harm inflicted. The following section focuses on the primary ecosystems impacted by the invasion of *A. conyzoides*.

### 5.1. Agricultural Ecosystem

*Ageratum conyzoides* is a devastating agricultural weed that affects nearly 36 crop species and is found in 46 countries [9]. It affects staple food crops as well as commercially important cash crops [31,78,79]. The establishment of permanent seed banks in the fields of the lower Shivalik range of the Himalayas due to heavy infestations of *A*. *conyzoides* had left them useless and abandoned [77,80]. Apart from being an agricultural weed, it is also known to host pests and pathogens of various crops. Different begomovirus-satellite complexes have been identified in *A*. *conyzoides* [81]. Reports show that *Ageratum enation*, a virus capable of infecting several important food crops, is hosted by this weed [82]. *A. conyzoides* also hosts tomato yellow leaf curl, cotton leaf curl, and okra enation leaf curl viruses [83,84] and, therefore, may act as an alternate source of infection in okra and tomato crops. The weed is a natural host for various aphid species that act as vectors for carrying papaya ringspot virus type P, a causal organism of papaya ringspot [85]. The Capsicum chlorosis virus was also reported to be hosted by *A. conyzoides* in the eastern regions of Queensland, Australia [21]. Infestations of the plant result in heavy monetary losses, particularly in the case of farmers with small land holdings [6].

### 5.2. Forest Ecosystem

Due to its shade-tolerant nature, *A*. *conyzoides* can maintain dense populations under tree canopies in forests [33,79]. For example, the understory of the tree plantations of *Acacia catechu*, *Eucalyptus* spp., *Pinus* forests, and mixed forests in the lower Himalayas of Himachal Pradesh (India) has been observed to be occupied by *A*. *conyzoides* [80]. It has been reported that *A*. *conyzoides* is among several exotic species that are posing a serious threat to the dense interior forests of Gandhamardan Hill Range, Odisha, India [86], the protected forests of Tripura [87], and a forest range in the village of Changki, Nagaland, India [88].

### 5.3. Grasslands and Rangelands

The dominance and ecological impact of *A. conyzoides* in grassland ecosystems have been observed by several researchers [89,90]. The fast-spreading stolons of *A*. *conyzoides* greatly enhance the weed’s potential to cover large areas of grasslands and rangelands, thus destroying native grasses and forbs and causing fodder shortages for livestock [4,90]. Furthermore, it reduces the carrying capacity of pastures and may lead to the disappearance of threatened and endemic species [4].

### 5.4. Soil Ecology

*Ageratum conyzoides* affects the soil chemistry, nutrient composition, and soil microbiota, thereby altering the environment of the invaded habitat [91]. A study reported a significant reduction in soil nitrogen and phosphorus in rice fields due to weed infestation [78], whereas other reports suggested that weed residues have enriched the soil nutrient content [31,92], despite their negative effects on associated crop species [31]. The weed modifies the soil environment through root exudation by mobilizing or chelating nutrients and, in turn, disturbing the natural soil composition [36].

### 5.5. Biodiversity

The ability of the weed to occupy available niches has reduced the availability of habitats for the local flora, which affects the biodiversity of the invaded areas [79]. *A*. *conyzoides* can easily outcompete medicinal-rich plants [4]. The weed has affected the biodiversity components of the invaded localities in the lower Himalayas by replacing native grasses and economically important herbs and creating homogenous stands [93].

### 5.6. Humans and Livestock

As a medicinal plant, *A*. *conyzoides* has limited uses due to its toxicity. *Ageratum conyzoides* at 500 and 1000 mg kg^−1^ can stimulate hematological disorders and may also affect the liver and kidneys in humans [94]. It can also cause dermatitis, nausea, bronchitis, and asthma. A study showed that it is one of the most common pollen allergens affecting patients with allergic rhinitis [95]. If livestock feeds on the plant due to a scarcity of fodder or immature taste buds, it can cause shivering, a very high fever, the production of bitter milk, anorexia, diarrhea, ulceration, or even lethal toxicity under extreme conditions [96].

The unchecked spread of *A. conyzoides* may have serious ecological and economic implications in different ecosystems, the magnitude of which is still not clearly known. Therefore, it becomes imperative to develop and implement appropriate management strategies to restrict its spread and mitigate its impact in an efficient, cost-effective, and eco-friendly manner.

## 6. Control and Management

Various methods have been proposed and practiced by agronomists and natural resource professionals for weed control. In this section, the most commonly and successfully used methods, in addition to their pros and cons, are considered.

### 6.1. Physical Methods

Physical control methods include uprooting, burning, or cutting (using blades, shrub masters, etc.), depending on the intensity of spread, the size of the area infected, and the stage of the weed that is being removed. However, this cannot be a feasible option if the area involved is large and is only a short-term solution if the plant has already set seeds [97]. Furthermore, the dangers of health problems, soil contamination by burning, and the likelihood of its re-emergence are also present [98].

### 6.2. Cultural Methods

In agricultural systems, the density of *A*. *conyzoides* can be reduced by using conservation tillage systems, and the residues left can also be used as mulch to enhance soil fertility [99]. Since the weed cannot germinate under anaerobic conditions, flooding the field for a short time can help control its infestations [100]. The density of *A*. *conyzoides* is best controlled by passing a wheel hoe at regular intervals along with hand weeding, or by using the stale seedbed technique along with inter-cultivation [101].

### 6.3. Chemical Methods

Both pre-emergence (oxadiazon, atrazine, oxyfluorfen, diuron, methazole, simazine, etc.) and post-emergence (glyphosate, 2, 4-D, etc.) herbicides are used to control infestations of *A*. *conyzoides*. In agroecosystems, the selection of the herbicide usually depends on the host crop species. Unlike grassy weeds, *A*. *conyzoides* appears late in maize fields and, therefore, is better controlled by a post-emergence spray of atrazine [102]. However, these herbicides pose environmental dangers; cannot control the regeneration of plants from root stumps, runners, suckers, stolons, or the seed bank present in the soil; and may result in the production of resistant species [97,98].

### 6.4. Biological Methods

The use of natural plant products, e.g., parthenin extracted from *Parthenium hysterophorus* and volatile essential oils from *Callistemon viminalis*, has been found to be effective in controlling *A*. *conyzoides* [103,104,105]. Monoterpenes, such as cineole and citronellol, which are found in members of the citrus family, also have potential for the management of *A. conyzoides* [106]. As a cover crop and mulch, both *Chromolaena odorata* and *Mikania micrantha* showed allelopathic properties against the growth of *A*. *conyzoides* [107]. Layering the soil with residues of *Dicranopteris linearis* inhibited the growth of *A*. *conyzoides* seedlings [108]. Even endophytic actinomycetes isolated from different plants are rich sources of herbicidal metabolites and can be employed against the weed [109].

Pathogens, insects, and nematodes have been introduced from the native ranges of *A*. *conyzoides* to serve as natural enemies of the weed; none, however, have proven effective. These were found to be polyphagous and, thus, had the strong potential to become pests of many other useful plants. Therefore, these are generally not recommended as a management strategy for the weed.

### 6.5. Field and Crop Management

Knowledge of the patterns of weed emergence can prove advantageous in planning their management. Herbicidal applications can be more beneficial if an understanding of the plant developmental cycle is achieved [110]. After harvesting, instead of being left fallow, crop fields can be used to grow legume crops or other useful plants to occupy the empty niche. This also applies to the areas where the weed is removed to prevent reinfestation. Palisade grass (*Urochloa brizantha*) cropping at regular intervals and sorghum intercropped with congo grass (*Brachiaria ruziziensis*) have also been reported to decrease the seed bank of *A*. *conyzoides* [111]. Growing sweet potato (*Ipomoea batatas*) varieties in fields dominated by *A*. *conyzoides* has been shown to reduce their growth, biomass, and yield traits [112]. Furthermore, vermicomposting is another viable, eco-friendly, economical, and proven solution for the effective and on-site management of *A. conyzoides* [79]. The compost of *A. conyzoides* prepared using a rotary drum composter was also found to be nutrient-rich and non-toxic and can be used effectively as a soil conditioner [91].

Though our understanding of the ecology and management possibilities of *A. conyzoides* has improved considerably, challenges remain to control its spread. We recommend a six-step management guide provided by Batish et al. [97] to manage *A*. *conyzoides*. It begins with targeting an infested area, followed by (a) the compilation of all the necessary information on the weed, (b) understanding weed biology, mode of spread, and invasive characteristics, (c) estimating its monetary, ecological, and socio-economic impacts, (d) creating awareness among local people, (e) taking appropriate control measures, and (f) devising preventive measures to avoid the re-emergence of the invasive weed.

Integrated weed management is the best approach to monitoring and regulating any weed, including *A*. *conyzoides*. Individual strategies involving physical, chemical, and biological methods have failed to provide long-term control, but an integrated approach utilizing all these techniques may prove to be relatively successful [91,113]. A suitable set of control measures (taking into consideration the extent and intensity of infestation) should be selected and employed with the involvement of government and non-governmental organizations, researchers, conservation managers, agriculturists, and local people. The least infested areas should be targeted first, followed by those with dense infestations. The waste accumulated may even be utilized for biogas or compost production [79,91,114]. With the help of the public, the further re-emergence and spread of the weed should be checked. Consistent follow-up work with the participation of both higher authorities and local communities is essential for the sustainable management of *A*. *conyzoides*. Furthermore, promoting its utilization at both commercial and non-commercial scales can offer economically viable and beneficial options for its management.

## 7. Conclusions and Way Forward

*Ageratum conyzoides* exhibits diverse biological characteristics that contribute to its significance both in medical and socio-economic contexts while also amplifying its invasive tendencies. This review drew attention to its expanding global distribution, the biological characteristics enabling its invasive success, the resulting ecological impacts of infestation, and the management options considered so far. In addition, the economic value of the species, along with traditional and modern applications, was highlighted. This discussion also sought to identify lesser-explored aspects and knowledge gaps in the ongoing research to suggest potential areas for future research. While the pharmacological and industrial applications of the plant have received considerable attention, the consequent impacts of its spread remain relatively unexplored. It is important to cover any possible lacunae in our understanding of its invasive behavior to strengthen its management at different levels. Additionally, the relationship between its toxicity and bioactivity, which is crucial for validating its medicinal properties, has not been adequately addressed. Given the invasive nature of *A. conyzoides* and its diverse biological activities, there is considerable anticipation for its potential as a botanical drug or pesticide. Consequently, there is a need to enhance *A. conyzoides*-based products using advanced tools and technologies to fully harness their therapeutic and pesticidal properties.

## Data Availability

Not applicable.
